# Long-Term Services & Supports available to Older individuals with Intellectual and Developmental Disabilities

**DOI:** 10.1093/geroni/igaf122.2326

**Published:** 2025-12-31

**Authors:** Akwasi Adjei Gyimah, Eric Frimpong, Samuel Asante, Mathias Adjei

**Affiliations:** Miami University Scripps Gerontology Center, Oxford, Ohio, United States; University of Massachusetts Boston, Boston, Massachusetts, United States; Northeastern State University, Tahlequah, Oklahoma, United States; Miami University, Oxford, Ohio, United States

## Abstract

This study investigates the Long-term Services and Supports (LTSS) provided to individuals with intellectual and developmental disabilities (IDD) at two selected Special Schools. The primary objective was to enhance our understanding of services available to older adults with IDD from the perspective of the headteacher, housemothers, family members of IDD, and older adults. Using a robust qualitative case study approach, an in-depth interview was conducted to gather the insights of participants on services available to older individuals with IDD. A purposive sampling technique was employed, and interviews were conducted with all participants. The interview guide for older adults with IDD had graphical images to enhance comprehension and easy answering of questions. The study used the cumulative advantage and disadvantage model as a conceptual framework. The findings showed that family members of older adults with IDD have little influence in helping their loved ones achieve independence through some LTSS programs. The burnout for the housemothers was severe and a faith-based mechanism was the only coping strategy employed. Older adults with IDD felt more comfortable and included in daily activities in school than at home. The home environment was tagged as a place of stigma and stereotypes for them and felt like an unwanted environment. The government officials felt they had no academic knowledge on how to help older adults in the Special schools. This study makes a significant contribution to the academic discourse on demonstrating how to make LTSS more responsive to the needs of older individuals with IDD.

